# Development and Evaluation of an Ultrasonic Humidifier to Control Humidity in a Cold Storage Room for Postharvest Quality Management of Dates

**DOI:** 10.3390/foods10050949

**Published:** 2021-04-26

**Authors:** Maged Mohammed, Nashi Alqahtani, Hamadttu El-Shafie

**Affiliations:** 1 Date Palm Research Center of Excellence, King Faisal University, Al-Ahsa 31982, Saudi Arabia; nalqahtani@kfu.edu.sa (N.A.); helshafie@kfu.edu.sa (H.E.-S.); 2 Agricultural Engineering Department, Faculty of Agriculture, Menoufia University, Shebin El Koum 32514, Egypt; 3 Department of Food and Nutrition Sciences, College of Agricultural and Food Sciences, King Faisal University, P.O. Box 420, Al-Ahsa 31982, Saudi Arabia; 4 Department of Crop Protection, Faculty of Agriculture, University of Khartoum, Shambat 13314, Sudan

**Keywords:** ultrasonic transducer, microcontroller, physicochemical characteristics, cold storage, microbial contamination, stored product, fruit quality

## Abstract

Dates are subjected to postharvest losses in quality and quantity caused by water loss, fermentation, insect infestation, and microbial spoilage during storage. Cold storage is the main element in the postharvest quality management used for fruit preservation. Although cold storage is used for dates, precision control of the relative humidity (RH) using ultrasonic applications is not used thus far, or it is applied to other fruits on a small scale. Therefore, we designed and constructed an ultrasonic humidifier (DUH) for RH control in the cold storage room (CSR) of dates. The optimum air velocity of 3 m s^−1^ at the outlets of the DUH ducts produced a mist amount of 6.8 kg h^−1^ with an average droplet diameter of 4.26 ± 1.43 µm at the applied voltage of 48 V and frequency of 2600 kHz of the transducers. The experimental validation was carried out by comparing a CSR controlled with the DUH with two conventional CSRs. The three tested CSRs were similar in dimensions, cooling system, and amount of stored dates. The time required for cooling 800 kg of dates in the controlled CSR from 25 °C to the target temperature of 5 °C was approximately 48 h. The DUH precisely controlled the RH at the maximum RH set point of 80% in the tested CSR at 5 °C. The controlled RH at 80% has a positive impact on the physicochemical characteristics of the stored dates. It significantly reduced the weight loss of the fruits and preserved fruit mass, moisture content, water activity, firmness, and color parameters. However, no significant effect was observed on fruit dimensions, sphericity, and aspect ratio. The microbial loads of mesophilic aerobic bacteria, molds, and yeasts fell within the acceptable limits in all tested CSRs. Both stored date fruits and artificially infested dates showed no signs of insect activity in the controlled CSR at the temperature of 5 °C and RH of 80%. The DUH proved to be a promising technology for postharvest quality management for dates during cold storage.

## 1. Introduction

The global demand for food is expected to increase in the upcoming years due to the world population increase [1,2]. However, substantial food losses and waste occur during postharvest handling, processing, storage, distribution, and consumption [3]. The use of innovative technologies to preserve food will reduce postharvest losses that eventually lead to food insecurity [4]. According to the standards in [5], freedom from live insects, dead insects, eggs of insects, mites, and pathogenic microorganisms is one of the most important quality indices of dates [6]. Dates are subject to microbial infection during harvesting and handling processes through potential spoilage microorganisms such as bacteria, yeasts, and molds. Stored fruits under unsuitable storage conditions of temperature and humidity are accompanied by microbial spoilage [7,8]. The major microorganisms causing pathological disorders of dates through fermentation and decay include yeast of the genus *Zygosaccharomyces*, *Acetobactor* bacteria, and fungi of the genera *Aspergillus*, *Alternaria*, and *Penicillium* [6,9]. The major postharvest pests include the date moth *Cadra cautella* and the saw-toothed beetle *Oryzaephilus surinamensis* [10,11,12]. The larvae of the date moth and larvae and adults of the saw-toothed grain beetle cause economic losses of stored dates in all date-producing countries [12,13,14]. There are a few options for the management of stored product insect pests and microorganisms such as cooling, ultrasonic and pulsed electric field applications, fumigation, and heat treatments after harvesting. Among these options, the use of cooling and cold storage remains of high potential [7,11,15,16,17,18].

The stored fruits are usually accompanied by water evaporation, respiration, and physiological changes due to the water losses which could cause heavy losses reaching up to 40%. Refrigeration or storage at low temperatures is the most common method used to increase the storage life of dates (Benkeblia, 2020). Therefore, dates, like most fruits, need to be stored at low temperatures after harvesting to slow down the insect activity and the growth of microorganisms responsible for quality deterioration. In addition, low temperature minimizes the vapor pressure between the product and the outer atmosphere, reducing the water loss from the stored date fruit [19]. Cold storage systems preserve the quality of agricultural products by delaying the ripening process, reducing moisture loss, and preventing microbial and insect spoilage [20]. The most critical factors in cold storage are relative humidity (RH) and temperature that extend the postharvest shelf life of fruits [21].

Food spoilage and the activity of aerobic bacteria, yeast, and fungi decrease in storage under a refrigerator cooling temperature of 5 °C compared with those stored at 25 °C for 12 months [22]. On the other hand, high-humidity conditions are needed in order to prevent water loss to maintain the characteristics of the fruit during low-temperature storage. As most fungi cease to grow under RH conditions of less than approximately 90% and a few fungi can grow at 85% RH, the RH of 90% is usually the most suitable for fruit storage [19]. The atmospheric humidity and temperature must be adjusted at optimal levels anywhere in the CSR. Different humidity and temperatures at various levels in the stores cause losses in product quality [23]. Evaporative water from an unwrapped agricultural product such as fruit, vegetables, and others causes direct losses of the product and limits its display life through dehydration and deterioration. Thus, humidification systems can offer a solution to reduce these losses [24]. During the postharvest cold storage of fruits, water loss could be prevented by controlling high RH, ideally between 90 and 95% [18]. Previous studies have shown that humidification of agricultural products such as fruits and vegetables had no adverse effects on microbial quality [25]; this may be due to the use of water disinfected by ozone. In another previous study on broccoli, the researchers mentioned that the misting by chlorinated tap water in CSRs led to reduced bacteria growth [26].

Generally, humidification systems in the cold storage rooms (CSRs) of food increase the water amount in the air to reduce the difference between water vapor pressures at the food surface and in the air. This difference is the reason behind the evaporation of water in food. Ultrasonic humidifier systems in refrigerated food and living environments utilize excited ultrasonic transducers immersed in baths of water to make a fine mist. Using different approaches, atomizing systems deposit small water droplets directly onto the food to replace water loss by evaporation [24].

Ultrasonic applications are promising technologies for postharvest humidification and processing of agricultural products. Ultrasonic atomization is defined as liquid disintegration to obtain droplets in the surrounding air by an ultrasonic transducer [27,28,29,30,31,32]. It occurs due to the competition between cohesive and destructive forces on the liquid’s surface, leading to disturbances and fluctuations in the liquid and finally producing a suspension that can be defined as an aerosol, spray, fog, or mist [33]. Generally, ultrasonic humidifiers’ operating principle depends on converting the electrical energy into periodical mechanical vibration by piezoelectric transducers and horn vibrations at high frequencies. If the ultrasonic waves have enough energy to overcome the water surface tension, then droplets will be generated from the water top surface. The droplet size produced by the ultrasonic humidifier depends on the frequency of the applied ultrasonic transducer. Therefore, it is easy to generate droplets in the micron size range using driving frequencies in the range of 2500 to 2600 kHz [15,28]. When the vibrating amplitude of the transducer surface is increased to the level of collapse and instability of the free liquid surface above it, the droplets will be ejected away from the water surface into a mist form. The droplet size of the mist is dependent on the frequency of vibration, water depth above the piezoelectric transducers, and the viscosity, surface tension, and density of the fluid [34,35]. Additionally, the atomized droplets number is greater when increasing the ultrasonic frequency at the same fountain shape. The ultrasonic transducer’s fountain is shaped at the water surface when the sufficient acoustic pressure is more than the atmospheric pressure. The ultrasonic humidifier (UH) produces tiny droplets, nearly uniform (monosized), with lower velocities than droplets generated from other atomizer types. The UH has a low cost and low energy consumption. Due to these droplet properties, ultrasonic humidifiers are suited to and very useful in medical applications, combustion, and humidification applications and can be efficiently used in spray drying [35,36].

Understanding food characteristics is necessary to determine the optimal solution for food processing, preservation, and storage problems [37,38,39,40]. Density, porosity, and shrinkage represent agricultural products’ structural properties due to their significant impact on food quality, texture, and food process design [37,41,42]. The most critical characteristics of foods are moisture content and water activity because water is an essential constituent. There is a relationship between the moisture contained in a food and its relative tendency to spoil. The relative vapor pressure (vapor pressure relative to that of pure water) is termed water activity (a_w_). A food’s water activity is related to its stability deterioration due to microorganisms’ growth [37,43]. The water sorption properties are essential in predicting a material’s physical state at various conditions because most phase transitions and structural transformations are influenced by water [44,45,46,47]. The moisture content of date palm fruits is the limiting factor affecting the shelf life and storage stability [47]. The water activity indicates a combination of water–surface interactions and water solubility and capillary forces, and it increases with pressure and temperature increases. Water activity controls the microbial stability, chemical stability, hardness, and compaction of the stored products. Therefore, investigators are interested in studying food moisture content and water activity due to the great significance in determining the physical and sensory properties, processes, and shelf life of foods [37,39,43].

Although modern cold storage is used in date fruit storage, accurate control of the RH is empirically used at a small scale or in the laboratory. In this respect, few published studies are available describing the impact of RH on the quality of dates inside refrigerated stores. The RH of the airflow within the cold store is increased by spraying water as a fine mist controlled with a humidistat. Wetting the floor of the cold storage or using water vapor also increases RH. The main objectives of this study were to design an ultrasonic humidifier for controlling the RH in the cold storage room of dates, to evaluate the performance of the designed system, and to study some physicochemical characteristics of dates and potential contamination before and after cold storage.

## 2. Materials and Methods

### 2.1. Description of the Designed Ultrasonic Humidifier

**Figure 1:**
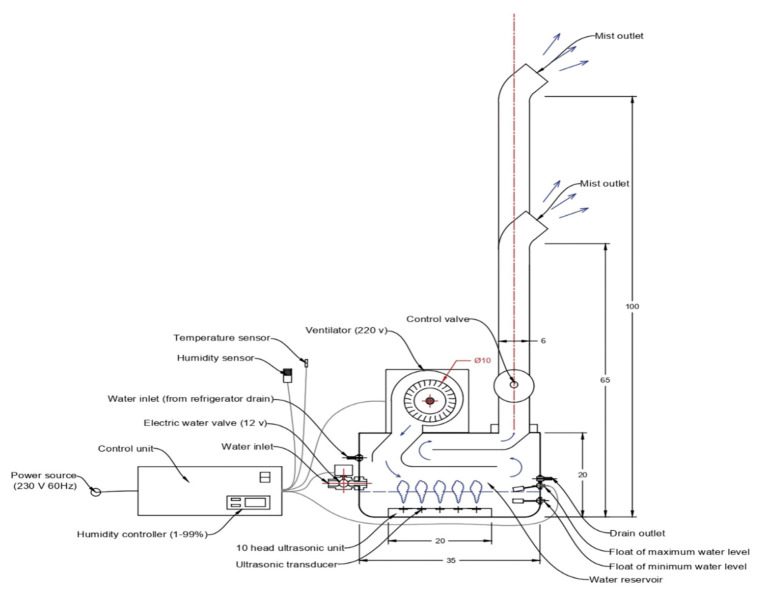
Schematic diagram of the designed ultrasonic humidifier showing the different parts (dimensions in cm).

**Figure 2:**
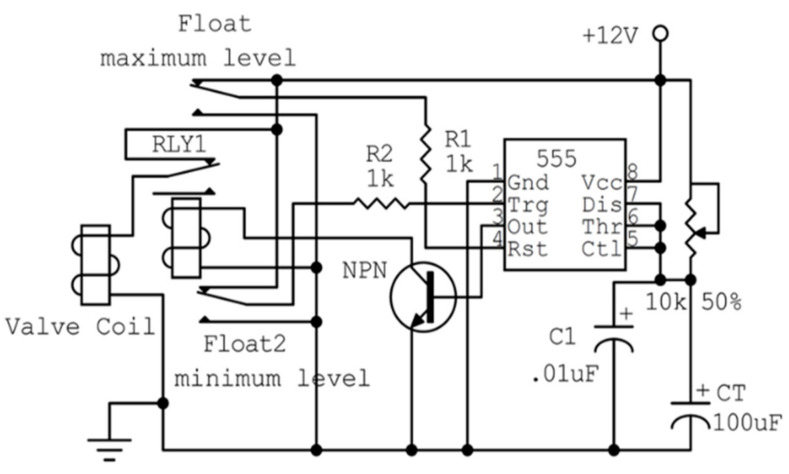
Circuit diagram of water level control in the humidifier reservoir.

We designed and constructed an ultrasonic humidifier to control the RH in the cold room to avoid the effects of different humidity levels on the quality of the stored dates. The ultrasonic humidifier consisted of a reservoir, ultrasonic transducers, a ventilator, and a control unit, as shown in Figure 1. The reservoir was a rectangular stainless steel tank of 0.20 × 0.20 × 0.35 m. The maximum amount of available water that could be turned into droplets by the ultrasonic transducers was 2.5 L. The water is drawn from a tank containing ozonated water to the humidifier reservoir by a small pump (12 V) via an electric solenoid valve with an inlet diameter of 0.05 m. After the reservoir is filled, the water level circuit closes the electric valve and the pump (Figure 2). A stainless steel float was connected with the circuit as a sensor. The water level circuit consisted of 2 capacitors, 2 resistors, a timer (Ne555n, Shenzhen Jin Da Peng Technology Co., Ltd., Guangdong, China), a 12 V relay, and an NPN (Negative-Positive-Negative) transistor (SL 100 NPN, Renesas Electronics Corporation, Tokyo, Japan). The ultrasonic transducer unit contained ten transducers installed at the central position of the humidifier reservoir bottom. The transducers’ ultrasonic frequency was 2600 kHz, the resonance impedance was 2 Ω, and the operating water temperature of the transducer unit range was 0 to 50 °C. The water height above the ultrasonic transducers ranged from 0.02 to 0.04 m. The water depth was adjusted at 0.03 m above the ultrasonic transducers to generate the water droplets efficiently. Droplets with a large size are dropped back into the humidifier reservoir through two stainless steel covers, which are fixed above the transducers. The generated droplets are diffused in the atmosphere of the CSR by the forced air through two vertical ducts made from plastic polyvinyl chlorides (PVC) with a diameter of 0.06 m. The height of the first duct from the bottom to the mist outlet was 100 cm and the height of the second duct was 65 cm.

**Figure 3:**
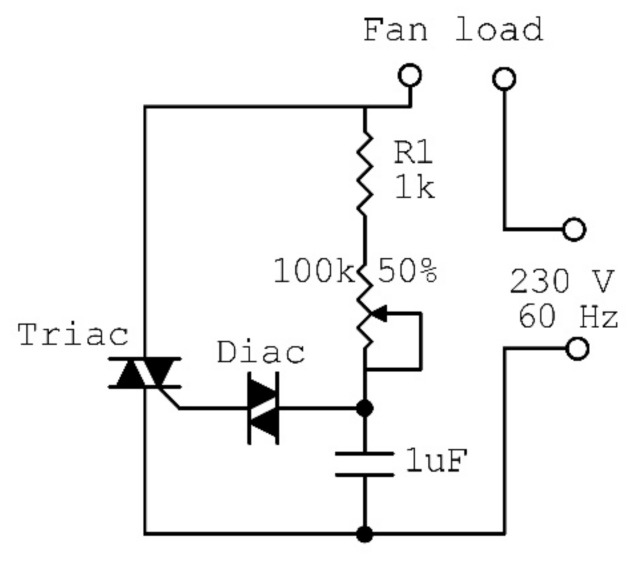
Circuit diagram of fan speed control.

The fine droplets were dispersed into the mist area in the reservoir, where mists were dispersed into the external air via a ventilator (model: RBPT12-14B, Zhejiang Lide Electric Co., Ltd., Wenzhou, China) through the mist ducts. The ventilator’s flow rate ranged from 0 to 2.5 m^3^/min, the rated inputted voltage was 220 V/60 Hz, and the maximum power consumption was 22 W. The ventilator flow rate was adjusted by controlling the fan speed using the electronic regulator circuit (Figure 3). The circuit consisted of a 1 KΩ resistor (R1), 100 KΩ variable resistance (R2), 1 capacitor of 0.1 µF (C1), a TRIAC (BT136, Renesas Electronics Corporation, TOKYO, Japan), and a DIAC (DB3, Renesas Electronics Corporation, TOKYO, Japan). The RH was adjusted using a high-precision humidity controller (model: MH13001 with a humidity sensor model: INS121, Shenzhen Electronic Co., Ltd., Guangdong, China). The measuring range of the controller ranged from 1 to 99%. The humidifier power supply’s input voltage was 230 V (60 Hz). The maximum output power was 250 W, the output voltage ranged from 36 to 50 VDC, the maximum output current was 5 A, and the working temperature was −1 to 55 °C.

### 2.2. Experimental Setup

**Figure 4:**
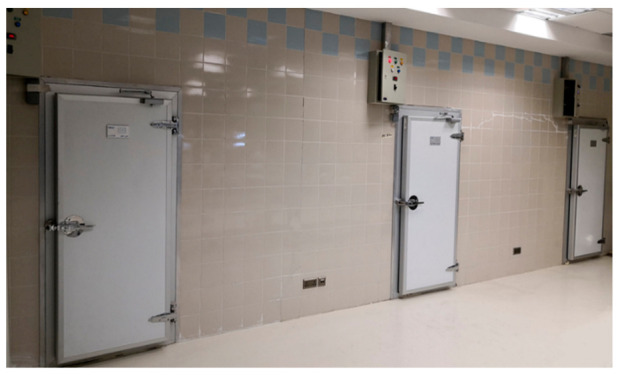
The three experimental cold storage rooms.

The experiment was carried out using three CSRs installed at the Date Palm Research Center of Excellence, King Faisal University, Saudi Arabia. These cold rooms were intended to cool and store dates produced from the center’s date palm fields, Figure 4. Each CSR consisted of thermally insulated walls, an evaporator, a condensing unit, and a control panel. The cold room was made from polyurethane sandwich panels with a thickness of 0.10 m. The room capacity was 48.8 m^3^, and its internal dimensions were 5.8, 2.9, and 2.9 m for length, width, and height, respectively. The supply voltage of the evaporators was 380 V (3 phase, 60 Hz), and the fluid was R417 A (model: CAE3264, TS min/max was −40/38 °C, Carrier Global Corporation, New York, USA). The condensing unit was equipped with a refrigeration compressor (model: MTZ-2040, 400 V, 3 phase, 60 Hz, 6.74 kW, Danfoss, Trévoux Cedex, France). The control panel consisted of five breakers, two contractors, a 24 h timer, and a digital temperature controller (model: XR06CX 230 VAC, Dixell, Pieve d’Alpago, Italy) to control the fans’ operation, the evaporator, and the condensing unit. All CSRs were illuminated by 3 compact fluorescent light bulbs (the power of each one was 25 W). The temperature was set before the start of the experiments using the controller supplied in each control panel of the cold storage.

**Figure 5:**
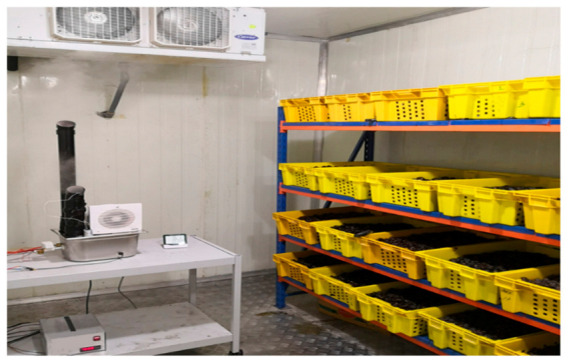
The experimental setup in the controlled cold storage room showing the designed humidifier and crates of stored dates.

The minimum and maximum set points of temperature were set at 4.5 and 5 °C for the first and second cold rooms and set at 0 and 1 °C for the third cold room. The ultrasonic humidifier was installed in the first cold room to control the RH, as shown in Figure 5, while the other rooms were left working traditionally without humidifiers. After the humidifier was installed in the cold room, the minimum and maximum set points of RH were set at 75 and 80% using the digital humidity controller. The RH set points were limited based on the water activity of the tested dates. This limit also does not allow the activity of microorganisms. For all cold rooms, the defrosting operation was carried out automatically as needed to reduce energy costs. The dates were stored after turning on the cold rooms when the inside temperature reached the target degree. The experiment duration was six months under the different atmosphere treatments. The physicochemical characteristics and microbial and insect contamination of stored dates were evaluated every month. Each cold room was packaged with a total weight of 800 kg of dates filled in 40 plastic crates. The condenser temperature was determined by installing an analog temperature sensor (Lm 35) on its surface. The temperature of dates inside the crates was also determined using three temperature sensors of Lm 35. The sensors were placed at a depth of 10 cm inside the top, middle, and bottom crates stored on the middle of the racks. The sensors of temperature and RH (DHT11) were placed inside the CSR to assess temperature and RH stability at the target set points. To study the effect of storage time on the stored date quality under RH control, the controlled CSR was compared with two traditional cold rooms with the same applied cooling system. One of the tested CSRs had the same temperature set points (4.5 and 5 °C), and the other one had a minimum and maximum set point of 0 and 1 °C, respectively.

### 2.3. Tested Date Fruit

The date fruit (*Khalas cv*.) used in this study was obtained, in season 2019, from the farms of the Date Palm Research Center of Excellence at the Agricultural Training and Research Station, King Faisal University, Saudi Arabia (Latitude: 25.27 °N, Longitude: 49.71 °E). Dates were packaged after harvesting in perforated plastic crates with an internal size of 0.48 × 0.36 × 0.23 m. Each crate was filled with approximately 20 kg of date fruit.

As date fruits are harvested while hot, they are susceptible to quality loss if directly stored after harvesting. If dates are stored at a high temperature, they will saturate the air inside the CSR. When the saturated air cools, the water will condense on the surface of the stored dates, and the stored dates will be susceptible to fungal or bacterial infection. Therefore, decreasing the initial temperature of dates must be carried out before cold storage. To avoid this problem, we decreased the initial temperature of dates from 38 to below 25 °C before storage for 48 h, and then 40 crates with a total weight of 800 kg were used for each CSR.

### 2.4. Measurements

#### 2.4.1. Droplet Measurements

The average droplet diameter was predicted based on the ultrasonic frequency of the applied transducer (2.6 × 10^6^ Hz), water density (999 kg m^−3^ at 5 °C), and surface tension (7.49 × 10^−2^ N m^−1^) using the following equation [48,49]:

(1) $$D_{d}=A\times \sqrt[{3}]{\frac{8\times \pi \times \sigma }{\rho \times f^{2}}}$$

where $D_{d}$ is the average droplet diameter (m), A is a constant (A = 0.34), *σ* is the water surface tension (N m^−1^), ρ is the water density (kg m^−3^), and *f* is the ultrasonic frequency (kHz).

**Figure 6:**
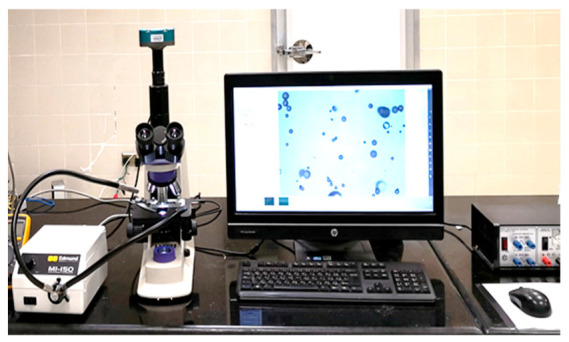
An image processing system for measuring the diameter and projected area of droplets produced by the designed ultrasonic humidifier. The system consists of a microscope with magnifications of 40×, a digital camera, a high-intensity illuminator, and an integrated desktop computer with the IS Capture software.

Dalmoro [33] used an optical microscope to characterize micro-droplets’ size produced by an ultrasonic atomizer. In this study, the diameter of droplets and their actual projected area were also measured for DUH evaluation using an optical microscope. The produced droplets were captured on sampling slides in the cold room to measure the actual diameter and projected area of the produced droplets. The upper surface of the slides was coated with paraffin oil to prevent droplets from sticking to them. The paraffin oil slowed the evaporation of the captured droplets, and the droplets retained their spherical shape for 3 min. The food color (ingredients of used food color were water, color E133, preservative E211, and acidity regulator E330) was added to the water in the humidifier’s reservoir. Therefore, colored droplets were captured on sample slides. The slides containing the colored droplets were then placed on a microscope (model: XJS404, KOZO Optical and Electronical Instrument Co., Ltd., Nanjing, China) with magnifications of 40×, and pictures were captured with a digital microscope camera (Model: Tucsen, ISH1000, 10 MP, Fuzhou Tucsen Photonics Co., Ltd., Fujian, China) and a high-intensity illuminator (model: MI-150, EO Edmund Optics Inc., Barrington, NJ, USA) was used to adjust the light. The captured images were processed with IS Capture software Version: 3.6.8 and an integrated desktop computer (model: HP Compaq Elite 8300, China), as shown in Figure 6. The image processing was performed in the lab at 18 °C and RH of 45%. The average diameter and projected area for 30 droplets were calculated under each tested velocity by capturing the droplets on three different slides, and the average of ten droplets was randomly estimated from each slide. The produced droplets were measured under different air velocities at a distance of 10 cm along a straight line from the mist outlet in the cold room.

The produced mist amount was measured based on water weight loss in the DUH reservoir using an electronic balance.

#### 2.4.2. Temperature and RH

Due to the long storage period of dates and the possibility of malfunctions in the cooling system or the designed humidifier at this period, it was necessary to monitor the real-time atmospheric conditions of CSRs and to collect the data and store them in a laptop. These data were used for evaluating the performance of the control unit of the designed humidifier and the cooling systems. Therefore, the temperature (°C) and RH (%) in the tested CSRs were measured using precision centigrade temperature sensors (LM35, Texas Instruments Incorporated, Dallas, TX 75243, USA) and RH sensors (AMT1001, Robotics Pvt. Ltd., Gujarat, India). The temperature and RH data were recorded using a high-voltage LabJack (model: U3-HV, LabJack CO., LAKEWOOD, CO, USA), and ProfiLab-Expert software (ProfiLab-Expert, Version 4.0, ABACOM, Ganderkesee, Germany). We used the components available in the extensive parts library of the ProfiLab-Expert software for linking and processing the sensors’ signals. The library of ProfiLab-Expert included all required components, and we chose from them the lab jack device (UH3), 15 equation blocks to multiply each signal by the calibration factor, a table for data logging of the 15 sensors, and 15 test meters for real-time display of the temperature and RH. Two RH sensors and three temperature sensors were distributed in each CSR.

As a precaution during the experiment to avoid any data loss, the data were recorded at ten-minute intervals using a temperature and relative humidity data logger (model: UNI-T UT330C USB, UNI-TREND technology CO. LTD., DongGuan, Guangdong, China) and a temperature logger with a thermistor probe (model: Tinytag; TK-4023, Gemini Data Loggers, Chichester, West Sussex, UK). The thermal images in the cold storage room were captured using a thermal camera (model: FLIR T250, Merlin Lazer Ltd., Crowbridge, East Sussex, UK).

#### 2.4.3. Air Velocity

Air velocity distribution in the cold room and the humidifier’s outlets was measured using a hot wire anemometer with a real-time data logger (model: Omega HHF2005HW, OMEGA Engineering, Inc., Norwalk, CT, USA).

#### 2.4.4. Physicochemical Characteristics

The physicochemical characteristics of date fruit were determined to study the impact of RH controlled by a humidifier on date quality. The parameters were measured immediately after harvest, before storage, and during storage using 100 date fruits. The sampling design for dates in [5] was followed. A gross sample of 3000 g was randomly selected after harvest and was uniformly mixed before a sub-sample of 100 date fruits was randomly taken for subsequent measurements. Likewise, sampled fruits were selected randomly and taken from different locations during cold storage treatments.

- Moisture content

The date fruits’ moisture content was determined by drying a sample of 100 g at 70 °C under vacuum for 72 h using a vacuum-drying oven (model: LabTech, LVO-2041P, Korea). The moisture content was determined according to the standard methods of analysis of AOAC [50] using the following equation:

(2) $$M_{C}=\frac{\left(m_{I}-m_{d}\right)\;}{m_{d}}\times 100$$

where $M_{C}$ is the fruit moisture content dry base (d.b %), $m_{I}$ is the initial mass of the fruit (g), and $m_{d}$ is the fruit dry mass (g).

- Shape and size

The shape and size parameters of dates were determined using the method described by [12,40]. A digital camera (model: IXUS 185, Canon Inc., Tokyo, Japan) and an LED light source were used to capture the fruits’ image. The captured images were processed using an open source image processing software (ImageJ/Fiji 1.46, LOCI, University of Wisconsin, Madison, USA) to measure fruit dimensions, aspect ratio, and sphericity [12].

- Fruit color

The color measurements of date fruits were measured based on the CIELAB color space using a Hunter Lab color meter (model, Quest-45/0 LAV, Hunter Associates Laboratory, Inc, Reston, USA). Chroma, hue angle, and the color difference were calculated using the following equations:

(3) $$C=\sqrt{a^{2}+b^{2}\;}\;$$

(4) H=arc tanba if the value ≥0 

(5) H=arc tanba+360 otherwise

(6) $$\mathrm{CIELAB}\;\Delta E=\sqrt{{\left(L_{2}-L_{1}\right)}^{2}+{\left(a_{2}-a_{1}\right)}^{2}+{\left(b_{2}-b_{1}\right)}^{2}}$$

where the *L* value is the lightness, *a* and *b* are coordinates of chromaticity, *C* is chroma, *H* is the hue angle, *a* is the redness, *b* is the yellowness, and CIELAB ΔE is the color difference.

- Percentage of weight loss

The percentage of weight loss was estimated based on the difference between the mass of 100 date fruits before storage and after the target cold storage time. The following equation was used to estimate the weight loss:

(7) $$WL=\frac{\left(m_{I}-m_{t}\right)\;}{m_{I}}\times 100$$

where WL is the weight loss (%), $m_{I}$ is the initial mass of the date sample, and $m_{t}$ is the mass of the date sample (g).

- Total soluble solids

The total soluble solids (TSS) content of date fruits was determined using a laboratory refractometer (model: RFM 840, Richmond Scientific Ltd. Unit 9, Lancashire, UK) based on the standard analysis methods of AOAC [50].

- Firmness

The firmness of dates was measured based on the required pressure (N cm^−2^) for penetrating a stainless steel cylindrical probe with a diameter of 0.8 cm in the date fruit pulp. A fruit pressure tester (Model: FT 327) was used for measurements at a temperature of 25 °C.

- Water activity

Water activity ($a_{w}$) is a thermodynamic characteristic described as the ratio of the water vapor pressure in a controlled system to the pure water vapor pressure at the same condition. The measurements of $a_{w}$ were determined based on the standard analysis methods of AOAC [50] using a laboratory device (model: AquaLab Decagon Devices, Pullman Inc., Washington, DC, USA).

#### 2.4.5. Microbiological Analysis

Before and during cold storage treatment, the total microbial count was determined using the method described by [7,51]. Aerobic mesophilic bacteria were calculated using the pour plate method on Plate Count Agar (PCA CM0325, Oxoid Limited, Hampshire, UK). The plates were incubated for 72 h at 30 °C, and the counts were expressed as colony-forming units per gram (cfu g^−1^) for the tested samples. Yeasts and molds were cultured on potato dextrose agar plates (PDA CM0139, Oxoid Limited, Hampshire, UK) and incubated for 3 to 7 days at 30 °C. Coliforms were counted on Violet Red Bile Agar (VRBA, CM0107, Oxoid Limited, Hampshire, UK) using the pour. The plates were incubated at 37 °C for 48 h. Round, purple-red colonies (0.5 to 2 mm diameter) surrounded by purple-red haloes on VR-BA plates were counted as coliforms.

#### 2.4.6. Test Insects

The date moth *C. cautella* and the saw-toothed beetle *O. surinamensis* were reared in the entomology laboratory of the Date Palm Research Center of Excellence, King Faisal University, according to the methods described by [12,52], respectively. Both species were reared on dates of the cultivar ‘Khalas’ for at least three generations to ensure the test insects’ sound quality. Both test insects’ colonies were kept in an incubator with 50–60% RH at 29 ± 1 °C with 12 L: 12 D hours. Freshly laid eggs and instar larvae of *C. cautella* having different ages were used in the experiments. Khalas fruits were pitted (their seeds were removed) and cut longitudinally before artificially infested with larvae and eggs of *C. cautella*. Every ten fruits with one larva and five eggs (inside the fruit) were enclosed in a small fine nylon mesh bag (0.095 × 0.135 m) that allows gaseous exchange and prevents the escape of insects and were allowed to recondition or acclimatize to the new environment of the mesh bag before being introduced into the CSR. Likewise, unsexed *O. surinamensis* (30) adults were put in similar bags to those for *C. cautella*. They were allowed ten days in the rearing incubator for egg laying and the development of different life cycle stages. For each test insect, ten bags were then carefully inserted among the date lots in the cold room where dates were stored to determine whether the storage temperatures would affect their populations and activity. A sample of stored dates and three bags of artificially infested dates were selected randomly at the two-month interval and carefully inspected for insect activity. The mortality percentage of adult beetles in the treated dates was assessed according to the following formula:

(8) $$M_{insect}=\frac{N_{dead}}{N_{total}}\times 100$$

where $M_{insect}$ is the mortality percentage (%), $N_{dead}$ is the number of dead insects, and $N_{total}$ is the total number of tested insects. The mortality of larvae and eggs of *C. cautella* was determined with the same above formula for adults of *O. surinamensis*. For the eggs and larvae of *O. surinamensis*, the mesh bags were then kept in an incubator of the same temperature and relative humidity as the rearing conditions and were monitored for the presence of live larvae which also indicates the presence of would-be eggs.

### 2.5. Statistical Analysis

Data analysis was conducted by analysis of variance (ANOVA) using the statistical software of IBM SPSS (SPSS Statistics 24, SPSS Inc., Chicago, IL, USA). According to the Tukey test, multiple comparisons among the treatments’ means were conducted at a 0.05 level of significance.

## 3. Results

### 3.1. Humidifier Performance

**Figure 7:**
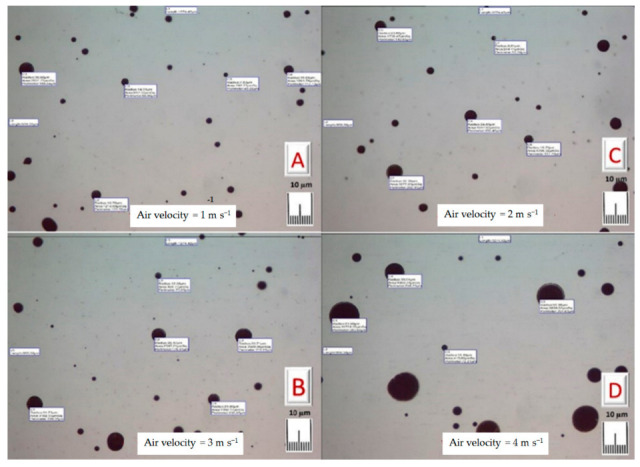
Images of the droplets captured using a digital camera of an optical microscope at different air velocities at the outlet of the ultrasonic humidifier ducts of 1 m s^−1^ (**A**), 2 m s^−1^ (**B**), 3 m s^−1^ (**C**), and 3 m s^−1^ (**D**) using 10 ultrasonic transducers with a frequency of 2600 kHz and an applied voltage of 48 V.

**Figure 8:**
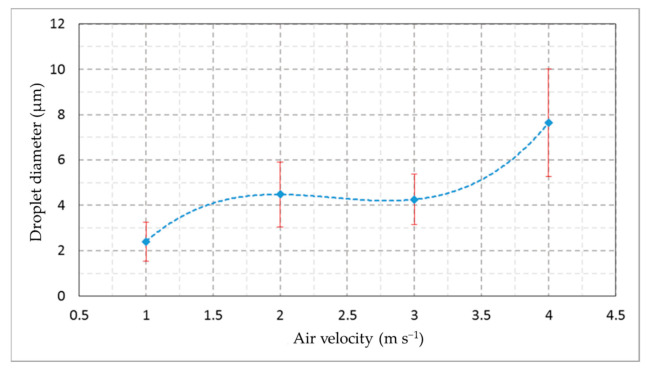
The relationship between air velocity at the outlet of the ultrasonic humidifier ducts and mean values of droplet diameter measured under RH of 80% and temperature of 5 °C.

Figure 7 shows the droplets’ images that were captured under different applied air velocities in the same position. The pictures indicate that the faster the air, the greater the size of the droplets. Figure 8 illustrates the diameter of droplets generated from the designed humidifier in the cold room conditions (80% RH and 5 °C temperature) with a constant water depth of 3 cm above the transducers. Table 1 shows the effect of air velocities of 1, 2, 3, and 4 m s^−1^ at the outlet of the ducts of the designed ultrasonic humidifier on the mean values of the droplets’ projected areas. The predicted droplet diameter and projected area were 2.22 µm and 3.79 µm^2^, respectively. The actual values of the droplet diameter and projected area were slightly higher than the predicted values that may be due to the adhesion of some of the droplets to each other. There was a significant difference within the mean droplet diameters and projected areas under the different applied air velocities (ANOVA, F_3, 29_ = 19.48, *p* = 0.000 and F_3, 29_ =11.96, *p* = 0.000, respectively). No significant differences were found between the mean diameters of the droplets at the air velocities of 2 and 3 m s^−1^. Likewise, there were no significant differences between the mean projected area of the droplets at the air velocities of 2 and 3 m s^−1^. This may be due to the large droplets’ transference being up with the air, which may fall into the humidifier reservoir again at lower values of air velocity. The high air velocity may sweep the droplets collected on the internal surface of the humidifier ducts. In this respect, Brizio [53] measured droplet sizes using an optical microscope and photographic and optic methods. He mentioned that there was no significant change between the methods with the power applied to the ultrasonic transducer.

In our experiment, the air velocity of 3 m s^−1^ at the outlet of the DUH ducts was applied to produce a mist amount of 6.8 kg h^−1^ with an average droplet diameter of 4.26 ± 1.11 µm. The suspended water droplets in the air did not negatively affect the room’s refrigeration. Additionally, the droplets reached every corner in the CSR homogeneously with the same temperature in the cold room. The humidity suspended in the air did not negatively affect the room’s refrigeration and did not freeze or condense on the room walls or the surfaces of stored dates.

### 3.2. Management of CSR Atmosphere

#### 3.2.1. Temperature

**Figure 9:**
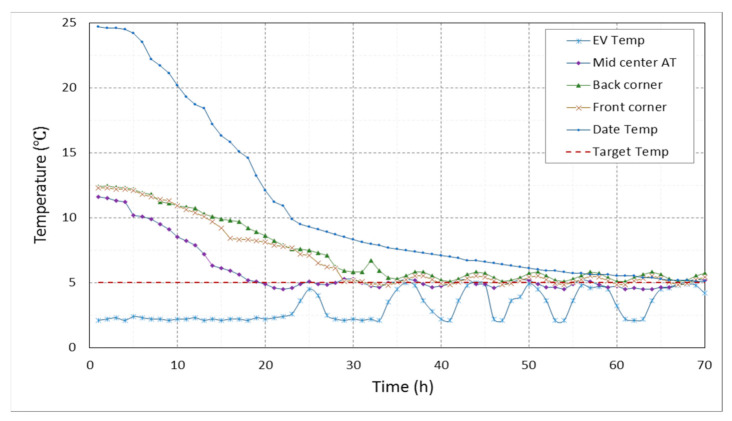
Temperature distribution in the controlled cold storage room for the evaporator (EV Temp), at the centerline of the cold room (Mid Center AT), at the corners behind the evaporator (Back corner), at the front corners (Front corner), and in the middle of date crats (Date Temp) at the target temperature (Target Tem) and 80% relative humidity.

Figure 9 shows the temperature variations inside the controlled cold room in front of the evaporator, in the center of the room, in the corners behind the evaporator, in the front corners, and in the middle crates of dates at the target temperature of 5 °C and RH of 80%. Each value in the chart describes the average temperatures of two different locations in the treatment chamber and date racks. The required time for cooling the centerline of the room to the temperature of 5 °C after dates were stored (800 kg with a temperature of 25 °C) was approximately 18 h. The time required for cooling the stored dates from the initial temperature to below 5 °C was approximately 65 h. Stability of the temperature in the two CSRs at the target temperature of 5 °C occurred after 70 h, and there was no significant difference between the rooms with controlled RH or not. The required time increased for the temperature of the third CSR until stability at the target temperature of 1 °C was achieved by approximately 12 h.

#### 3.2.2. Relative Humidity (RH)

**Figure 10:**
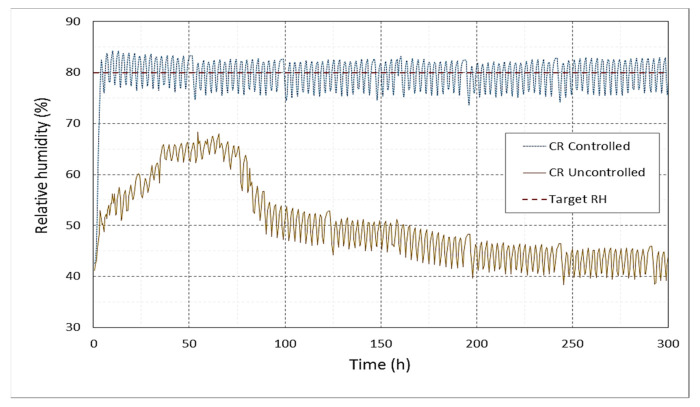
Relative humidity in the centerline of the controlled cold storage room (CR Controlled) and of the uncontrolled cold storage room (CR Uncontrolled) versus the target relative humidity of 80% (Target RH).

The designed humidifier achieved the target moisture content, as observed in Figure 10. A significant difference was observed between the two rooms by comparing the cold room with controlled RH and the traditional cold room. Although the RH increased in the first 50 h in the uncontrolled room and it reached stability at near 65% in about 40 h, a sharp decrease occurred after 75 h, and from 100 h, the decrease continued but more slowly. The decreasing RH is caused by the withdrawal of water from the cold room atmosphere, which flows through the air cooler and condenses, as a frost, on the air cooler’s surface. The frost on the evaporator’s surface is then removed thereafter by the defrosting unit of the evaporator. Defrosting is carried out automatically by feeding electrically generated heat. Despite the fact the defrosting process causes the withdrawal of water from the air of the room atmosphere, it is essential to prevent the excessive build-up of frost on the cooler surfaces to improve the heat transfer and optimize the cold storage operation system. Moreover, the automatic defrosting process creates free air circulation and improves the air cooler performance.

#### 3.2.3. Air Velocity

**Figure 11:**
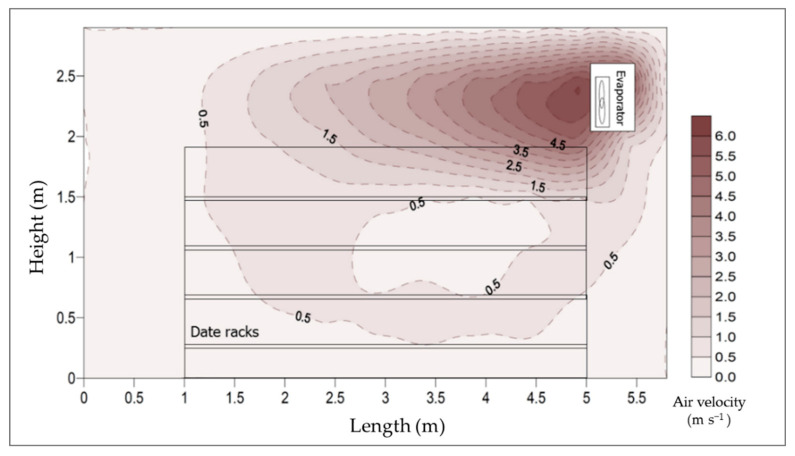
Vertical distribution of air velocities inside the cold storage room along the longitudinal centerline.

**Figure 12:**
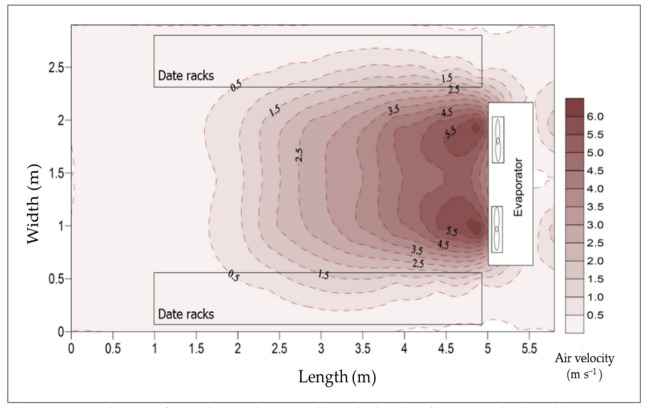
Distribution of air velocities horizontally at the height of 2.2 m inside the cold storage room.

A sufficient air movement was conducted in the cold room to avoid a heat transfer limitation on the thermal conduction because heat energy moves out of the stored product into the surrounding atmosphere. The air velocity in front of the racks in the CSRs was found to be 0.5 m s^−1^ after stability. Figure 11 shows the vertical distribution of air velocities inside the CSR along the longitudinal room centerline when the evaporator fans were running. In contrast, Figure 12 shows the distribution of air velocities horizontally at the height of 2.2 m.

### 3.3. Management of Physicochemical Characteristics of Stored Dates

#### 3.3.1. Physicochemical Characteristics of the Tested Dates

Table 2 shows the most critical physicochemical characteristics of the date fruits that were immediately measured after harvesting to study the changes that would occur during cold storage.

#### 3.3.2. Effect of RH and Storage Time on Date Fruit Quality

The most critical physicochemical characteristics of dates were measured every month during a storage time of 6 months. It was observed that the effects of different cold storage atmospheric conditions were significant on weight loss (ANOVA, F_2, 209_ = 208.5, *p* = 0.000), moisture content of fruits (ANOVA, F_2, 209_ = 211.5, *p* = 0.000), TSS (ANOVA, F_2, 209_ = 5.51, *p* = 0.005), mass of fruits (ANOVA, F_2, 209_ = 11.79, *p* = 0.000), water activity of fruits (ANOVA, F_2, 209_ = 34.07, *p* = 0.000), firmness of fruits (ANOVA, F_2, 209_ = 44.26, *p* = 0.000), CIELAB ΔE (ANOVA, F_2, 209_ = 6.43, *p* = 0.002), and the color parameter of *L*. There were no significant effects of the cold storage atmospheric conditions on mean fruit dimensions of length and diameter, sphericity, aspect ratio, and basic color parameters of *L*, *a*, and *b*.

The results show a highly significant effect of the storage time on weight loss (ANOVA, F_6, 209_ = 57.64, *p* = 0.000), moisture content of fruits (ANOVA, F_6, 209_ = 36.96, *p* = 0.000), mass of fruits (ANOVA, F_6, 209_ = 2.33, *p* = 0.034), firmness of fruits (ANOVA, F_6, 209_ = 7.41, *p* = 0.000), sphericity of fruits (ANOVA, F_6, 209_ = 11.93, *p* = 0.000), aspect ratio of fruits (ANOVA, F_6, 209_ = 3.74, *p* = 0.002), CIELAB ΔE (ANOVA, F_6, 209_ = 94.75, *p* = 0.000), and color parameters of *L* (ANOVA, F_6, 209_ = 53.78, *p* = 0.000), A (ANOVA, F_6, 209_ = 18.91, *p* = 0.000), *b* (ANOVA, F_6, 209_ = 16.64, *p* = 0.000), and *C* (ANOVA, F_6, 209_ = 26.23, *p* = 0.000). There were no significant effects of the storage time on fruit TSS, water activity, and the color parameter of *H*.

The effects of the interaction between the cold room atmospheric conditions and the storage time were highly significant on weight loss (ANOVA, F_12, 209_ = 7.49, *p* = 0.000), moisture content of fruits (ANOVA, F_12, 209_ = 8.21, *p* = 0.000), water activity of fruits (ANOVA, F_12, 209_ = 2.33, *p* = 0.008), CIELAB ΔE (ANOVA, F_12, 209_ = 1.91, *p* = 0.035), and the color parameter of *L* (ANOVA, F_12, 209_ = 1.988, *p* = 0.027). There were no significant effects of the cold room atmospheric conditions and the storage time on the length and diameter of fruits, fruit mass, fruit firmness, sphericity of fruits, aspect ratio, TSS, water activity, and color parameters of *a*, *b*, *H*, and *C*.

The data presented in Table 3 show a comparison between the mean values of the main physicochemical parameters of date fruits affected by the interaction between the cold storage conditions and the storage time. The effect of the controlled cold room was highly positive on the most important quality parameters of the studied date fruits at all stages of the storage time. From this table, it can be realized that there were significant differences between the mean values of weight loss, moisture content of fruits, water activity, lightness (*L*), and color difference of the date fruits stored in the three studied CSRs for different storage times (*p* < 0.05).

**Figure 13:**
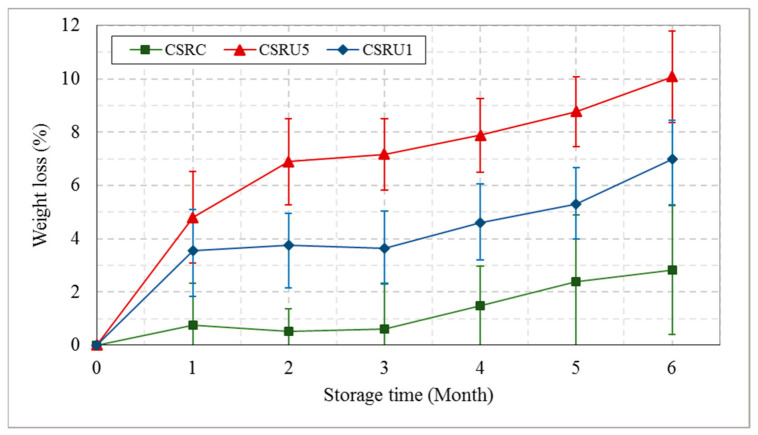
Comparison between the mean values of the weight loss of date fruits under different storage conditions of cold storage rooms and storage times. CSRC is the cold storage room with controlled RH at 80% and temperature of 5 °C, CSRU5 is the traditional cold storage room with a temperature of 5 °C, CSRU1 is the traditional cold storage room with a temperature of 1 °C.

It was observed that weight loss occurred from the first month for the date fruits in the two uncontrolled CSRs, as shown in Figure 13. There was virtually no weight loss for the date fruit samples in the controlled CSR under a controlled RH of 80%, 5 °C temperature, and air velocity of 0.5 m.s^−1^. The polynomial equations describing weight loss under the storage times for CSRs CSRC, CSRU5, and CSRU1 with coefficients of determination (R^2^) of 0.93, 0.96, and 0.99 were expressed in the following equations, Nos 1, 2, and 3, respectively, based on the observed data.

$$WL=0.007\;T^{3}+0.006\;T^{2}+0.179\;T+0.168\;WL=0.122\;T^{3}+1.158\;T^{2}+3.687\;T+0.255\;WL=0.142\;T^{3}+1.548\;T^{2}+5.855\;T+0.118$$

where *WL* is the percentage of weight loss, and *T* is the storage time.

Decreasing the RH in the traditional CSRs from 70 to 32% RH at 1 and 5 °C reduced the mean value of fruit mass from 8.79 to 7.83 g and from 8.77 to 8.11 g, respectively, at the same air velocity on the fruit surface (0.5 m s^−1^). Decreasing the RH in the CSRs also reduced the mean values of color parameters and increased the color difference. Increasing the temperature from 1 to 5 °C in the traditional CSRs increased the mean value of the color difference from 13.54 to 17.6 after the storage time of 6 months.

Hence, a significant impact of the ultrasonic humidifier was observed on decreasing the weight loss, color difference, and moisture content and maintaining the other studied quality parameters of dates compared with the traditional cold rooms for the studied storage time of 6 months.

Generally, the physicochemical characteristics were related to the three environmental boundary conditions of temperature, RH, and air velocity above the fruit surfaces. The RH is the most influential factor in the water loss of stored fruits [54]. Surface drying of stored date fruits by the air temperature and RH in CSRU1 and CSRU5 increased weight loss and led to color changes that were undesirable and resulted in a shorter shelf life of the fruits. Therefore, adjusting the temperature, RH, and air velocity in CSRC improved the optimum environmental boundary conditions, which had a direct impact on the quality and shelf life of the stored date fruits. Low temperatures slow down water losses and physicochemical changes, as well as the activity of microbial and insect life. However, the low-temperature effectiveness depends on the temperature applied, the initial quality attributes of the stored dates, and the insect and microorganism contamination present at harvest. Maintaining the optimum RH in CSRs at the prevailing temperature is very important in order to prevent either moisture uptake or drying of stored dates, except when they are packed in air-tight packaging [55]. The DUH in CSRC maintained the water amount in the air to reduce the difference between water vapor pressures at the food surface and in the air, as the difference in vapor pressure is a reason behind the evaporation of the moisture content in the stored dates or their uptake of water from the air [24]. In addition, the DUH produced small water droplets directly onto the stored date fruits to replace water loss by evaporation. The preservation of the moisture content of the stored dates resulted in preserving all the physicochemical characteristics.

### 3.4. Management of Microorganisms and Insects

The total counts of mesophilic aerobic bacteria, yeasts and molds, and coliforms before and during the storage period under CSR conditions were determined, as shown in Table 4. The average total count of mesophilic aerobic bacteria in the date samples before cold storage was 5.4 × 10^2^ cfu g^−1^, ranging from 6.4 × 10 to 6.2 × 10^2^ cfu g^−1^, while the average contamination with molds and yeasts was 7.8 × 10 cfu g^−1^ and ranged from 2.8 × 10 to 9.8 × 10 cfu g^−1^. No coliform contamination was detected in all tested samples before and during the cold storage in all evaluated cold storage conditions. There were no significant differences in the average total count of mesophilic aerobic bacteria in the three CSRs from the first month to the sixth month of storage compared to the initial contamination. The total number of yeasts and molds increased after six months, whereas the total number of yeasts and molds in the controlled CSR was 4.2 × 10^2^ cfu g^−1^ and, for uncontrolled CSRs under 5 °C and 0 °C, 2.8 × 10^2^ and 1.1 × 10^2^, respectively. It is worth mentioning here that the authors of [7,51] studied the microbial loads of four date cultivars, namely, *Khalas*, *Sukhary*, *Sugai,* and *Anbara*, collected from local markets and stored at 5 °C before testing (storage period was not specified). Their results indicated that the mesophilic aerobic bacteria load ranged from 2.0 × 10 to 2.1 × 10^3^, molds and yeasts ranged from 0.0 to 9.00 × 10^2^, and the load of coliforms ranged from 0.0 to 1.2 × 10^2^ cfu g^−1^. They mentioned that the microbial loads of mesophilic aerobic bacteria, molds, and yeasts fall within the acceptable limits according to the GSO technical regulation for completely prepackaged dates and Saudi standards, while the load of coliforms (1.2 × 10^2^ cfu g^−1^) had an unacceptable level. Recently, many technologies have been investigated to inactivate pathogenic microorganisms to avoid the spoilage of stored products at low temperatures, such as high pressures, pulsed electric field (PEF), ultrasonic, edible coatings, and modified atmosphere packaging [7,15,16,56,57]. One of these technologies could be applied before storing date palm fruits.

The periodical inspections of samples of both stored dates and artificially infested dates revealed no activity of insects. The mortality of all developmental stages of the two target species (*O. surinemensis* and *C. cautella*) was 100%. In this context, Aldosary [58] found that the infestation of dates by *O. surinamensis* increased the infection and fermentation by microorganisms. He thought that the beetle might help in the mechanical transmission of date decay-causing microorganisms. However, more investigations are needed to elucidate the interaction between beetle infestation and the development of molds responsible for the decay and fermentation of stored dates. The heat treatment of 55 °C for 60 min could be applied to date fruits immediately before cold storage as a safeguard against *C. cautella* infestation [11]. Storage of dates at 5 °C or below will control insect infestation and microbial spoilage as well [6].

## 4. Conclusions

To maintain the quality of dates during cold storage, they should be subjected to cooling immediately after harvesting. Surface drying of stored date fruits by the air temperature and relative humidity (RH) in traditional cold storage rooms increases weight loss and leads to color changes that are undesirable and result in a short shelf life of the fruits. In this respect, we designed and constructed an ultrasonic humidifier for controlling the RH in a cold storage room used to preserve dates. The validation of the designed humidifier was investigated based on the generated droplet size and its ability and precision in controlling the RH at the target value. The droplets produced by the humidifier had a unique performance in preserving the date fruit quality and reducing the water loss of the product during storage compared with similar conventional CSRs at 1 and 5 °C. Contamination by pathogenic microorganisms and infestation of insect pests were also prevented. The suspended water droplets in the air did not negatively affect the refrigeration system of the CSR, did not freeze or condense on the room walls or dates, and were spread homogeneously in the CSR. Finally, it can be concluded that the designed ultrasonic humidifier extended the shelf life of the stored date fruits without loss of their quality. Further investigations are needed to incorporate the designed humidifier with other modern preservation technologies for integrated postharvest quality management to extend the shelf life of date fruits.

## Figures and Tables

**Table 1:** Effect of air velocity at the outlet of the ducts of the designed ultrasonic humidifier on mean measured values of droplets projected under RH of 80% and temperature of 5 °C.

Flight Parameter	Air Velocity (m s^−1^)			
	1	2	3	4
Projected Area (µm^2^)	4.61 ± 3.9 ^c^	17.05 ± 10.59 ^B^	15.46 ± 8.09 ^B^	32.67 ± 1.7 ^A^

Figures within rows having similar letters are non-significant for each parameter at alpha values of 0.05.

**Table 2:** Mean values and standard deviations (St. Dev) of length (*L_f_*, mm), diameter (*D_f_*, mm), fruit sphericity (*S_f_*), fruit aspect ratio (*Ar_f_*), firmness (*F_f_*, N cm^−2^), water activity ($a_{w}$), mass (*M_f_*, g), moisture content (MC, %), total soluble solids (TSS, Brix), lightness (*L*), redness (*a*), yellowness (*b*), chroma (*C*), and hue angle (*h*, degree) of dates immediately after harvesting.

Value	Characteristic													
	*L_f_*	*D_f_*	*S_f_*	*Ar_f_*	*F_f_*	$a_{w}$	*M_f_*	MC	TSS	*L*	*a*	*b*	*h*	*C*
Mean	37.52	25.72	72.35	65.03	1.895	0.76	8.66	19.05	70.44	36.03	20.58	26.87	52.51	33.99
St. Dev.	1.92	2.47	4.61	5.40	0.22	0.07	0.71	1.24	1.08	4.94	4.22	5.30	4.97	6.03

**Table 3:** Comparison of the mean values ± standard deviation of weight loss (WL, %), moisture content (MC, %), water activity ($a_{w}$), lightness (*L*), color difference CIELAB (ΔE), and mass (*M_f_*, g) of the stored date fruits that were affected by different cold storage conditions and storage times before (C) and after storage from 1 to 6 months.

Storage Conditions	Storage Time	Characteristic				
		MC, %	*a_w_*	*L*	ΔE	*M_f_*
CSRC	C	19.33 ± 0.6 ^A^	0.77 ± 0.08 ^A^	40.2 ± 2.6 ^A^	0 ^H^	8.85 ± 0.73 ^A^
	1	19.15 ± 1.9 ^A^	0.78 ± 0.08 ^A^	39.6 ± 2.7 ^A^	1.09 ± 0.2 ^G–H^	8.83 ± 0.73 ^A^
	2	19.43 ± 1.0 ^A^	0.76 ± 0.05 ^AB^	38.9 ± 1.6 ^AB^	4.35 ± 3.6 ^E–H^	8.81 ± 0.75 ^A^
	3	19.26 ± 0.8 ^A^	0.78 ± 0.07 ^A^	36.8 ± 3.0 ^A–C^	6.37 ± 3.7 ^E–F^	8.82 ± 0.74 ^A^
	4	19.18 ± 1.0 ^A^	0.76 ± 0.07 ^AB^	35.4 ± 3.3 ^A–D^	13.3 ± 2.4 ^AC^	8.71 ± 0.62 ^A^
	5	18.89 ± 0.7 ^A^	0.78 ± 0.09 ^A^	31.2 ± 3.0 ^D–F^	14.5 ± 4.9 ^AB^	8.65 ± 0.80 ^A^
	6	18.86 ± 0.6 ^A^	0.76 ± 0.07 ^AB^	30.2 ±2.3 ^EF^	14.9 ± 4.4 ^AB^	8.59 ± 0.67 ^A^
CSRU5	C	19.20 ± 0.6 ^A^	0.77 ± 0.05 ^A^	40.1 ± 2.6 ^A^	0 ^H^	8.79 ± 0.72 ^A^
	1	15.22 ± 2.4 ^BC^	0.73 ± 0.06 ^A–C^	37.8 ± 2.6 ^A–C^	4.85 ± 3.6 ^E–H^	8.36 ± 0.68 ^A^
	2	14.48 ± 1.6 ^BC^	0.72 ± 0.07 ^A–C^	37.3 ± 2.3 ^A–C^	5.94 ± 3.8 ^E–G^	8.17 ± 0.59 ^AB^
	3	13.56 ± 2.0 ^B–D^	0.73 ± 0.06 ^A–C^	33.7 ± 3.9 ^B–E^	8.90 ± 3.2 ^C–E^	8.15 ± 0.56 ^AB^
	4	13.18 ± 2.3 ^CD^	0.63 ± 0.12 ^C^	31.1 ± 5.0 ^D–F^	12.4 ± 4.2 ^A–C^	8.12 ± 0.61 ^B^
	5	11.45 ± 1.4 ^D–E^	0.65 ± 0.12 ^BC^	29.7 ± 4.3 ^EF^	14.8 ± 4.9 ^AB^	8.01 ± 0.66 ^BC^
	6	10.30 ± 0.8 ^E^	0.63 ± 0.09 ^C^	28.4 ± 3.0 ^F^	17.6 ± 5.1 ^A^	7.83 ± 0.63 ^C^
CSRU1	C	19.06 ± 0.6 ^A^	0.77 ± 0.04 ^A^	39.8 ± 2.6 ^A^	0 ^H^	8.77 ± 0.72 ^A^
	1	15.69 ± 2.4 ^BC^	0.78 ± 0.08 ^A^	38.7 ± 3.0 ^A–C^	3.65 ± 3.0 ^F–H^	8.41 ± 0.72 ^A^
	2	15.88 ± 1.6 ^B^	0.78 ± 0.03 ^A^	35.9 ± 2.5 ^A–D^	5.66 ± 2.3 ^E–G^	8.39 ± 0.65 ^A^
	3	15.32 ± 2.2 ^BC^	0.79 ± 0.06 ^A^	33.6 ± 4.1 ^C–F^	7.91 ± 2.4 ^D–F^	8.40 ± 0.62 ^A^
	4	14.67 ± 2.3 ^BC^	0.79 ± 0.05 ^A^	33.5 ± 3.7 ^C–F^	8.81 ± 2.8 ^C–F^	8.37 ± 0.62 ^A^
	5	13.49 ± 2.0 ^B–D^	0.79 ± 0.05 ^A^	30.0 ± 3.1^EF^	12.0 ± 1.3 ^B–D^	8.26 ± 0.61 ^AB^
	6	11.91 ± 1.2 ^D–E^	0.80 ± 0.02 ^A^	29.3 ± 4.8 ^EF^	13.5 ± 2.5 ^A– C^	8.11 ± 0.66 ^B^

CSRC is the cold storage room with controlled RH at 80% and temperature of 5 °C, CSRU5 is the traditional cold storage room with a temperature of 5 °C, CSRU1 is the traditional cold storage room with a temperature of 1 °C. The same letter in each column of the measured characteristic indicates no significant differences at the 0.05 level (Tukey test).

**Table 4:** Comparison of the mean values ± standard deviation of the total counts of mesophilic aerobic bacteria, yeasts and molds, and coliforms before (C) and during the storage times from 1 to 6 months.

Storage Conditions	Storage Time	Microorganisms		
		Total Bacteria, cfu g^−1^	Molds and Yeasts, cfu g^−1^	Coliforms, cfu g^−1^
CSRC	C	5.40 × 10^2^ ± 1.71 × 10^2 A^	0.78 × 10^2^ ± 0.25 × 10^2 G^	n.d
	1	5.17 × 10^2^ ± 2.44 × 10^2 A^	1.31 × 10^2^ ± 0.54 × 10^2 E–G^	n.d.
	2	5.49 × 10^2^ ± 1.82 × 10^2 A^	1.86 × 10^2^ ± 0.66 × 10^2 C–G^	n.d.
	3	5.85 × 10^2^ ± 1.88 × 10^2 A^	2.37 × 10^2^ ± 0.92 × 10^2 B–F^	n.d.
	4	5.68 × 10^2^ ± 2.41 × 10^2 A^	2.84 × 10^2^ ± 1.03 × 10^2 BC^	n.d.
	5	5.92 × 10^2^ ± 2.06 × 10^2 A^	3.59 × 10^2^ ± 1.42 × 10^2 AB^	n.d.
	6	5.48 × 10^2^ ± 2.45 × 10^2 A^	4.20 × 10^2^ ± 1.63 × 10^2 A^	n.d.
CSRU5	C	5.40 × 10^2^ ± 1.71 × 10^2 A^	0.78 × 10^2^ ± 0.26 × 10^2 G^	n.d.
	1	5.34 × 10^2^ ± 2.41 × 10^2 A^	1.11 × 10^2^ ± 0.50 × 10^2 FG^	n.d.
	2	5.48 × 10^2^ ± 2.59 × 10^2 A^	1.47 × 10^2^ ± 0.72 × 10^2 D–G^	n.d.
	3	5.55 × 10^2^ ± 1.84 × 10^2 A^	1.84 × 10^2^ ± 0.68 × 10^2 C–G^	n.d.
	4	5.52 × 10^2^ ± 2.34 × 10^2 A^	2.59 × 10^2^ ± 1.04 × 10^2 B–E^	n.d.
	5	5.49 × 10^2^ ± 2.44 × 10^2 A^	2.79 × 10^2^ ± 1.15 × 10^2 B–D^	n.d.
	6	5.54 × 10^2^ ± 2.36 × 10^2 A^	2.80 × 10^2^ ± 1.31 × 10^2 B–D^	n.d.
CSRU1	C	5.40 × 10^2^ ± 1.71 × 10^2 A^	0.78 × 10^2^ ± 0.26 × 10^2 G^	n.d.
	1	5.36 × 10^2^ ± 2.45 × 10^2 A^	0.81 × 10^2^ ± 0.53 × 10^2 G^	n.d.
	2	5.47 × 10^2^ ± 2.63 × 10^2 A^	0.82 × 10^2^ ± 0.46 × 10^2 G^	n.d.
	3	5.49 × 10^2^ ± 1.86 × 10^2 A^	0.89 × 10^2^ ± 0.33 × 10^2 G^	n.d.
	4	5.42 × 10^2^ ± 2.67 × 10^2 A^	0.88 × 10^2^ ± 0.56 × 10^2 G^	n.d.
	5	5.43 × 10^2^ ± 2.92 × 10^2 A^	1.06 × 10^2^ ± 0.56 × 10^2 FG^	n.d
	6	5.49 × 10^2^ ± 2.31 × 10^2 A^	1.05 × 10^2^ ± 0.49 × 10^2 FG^	n.d

CSRC is the cold storage room with controlled RH at 80% and temperature of 5 °C, CSRU5 is the traditional cold storage room with a temperature of 5 °C, CSRU1 is the traditional cold storage room with a temperature of 1 °C, n.d. is non-detected. The same letter in each column of the measured characteristic indicates no significant differences at the 0.05 level (Tukey test).

## Data Availability

Not applicable.

## Systems Availability Statement

The designed ultrasonic humidifier and the cold storage rooms are available at the Date Palm Research Center of Excellence, King Faisal University, Al-Ahsa 31982, Saudi Arabia.
